# Glycolysis Changes in Alloreactive Memory B Cells in Highly Sensitized Kidney Transplant Recipients Undergoing Desensitization Therapy

**DOI:** 10.3389/ti.2024.13029

**Published:** 2024-07-08

**Authors:** Johan Noble, Lara Cabezas, Aurelie Truffot, Lucile Dumolard, Thomas Jouve, Paolo Malvezzi, Lionel Rostaing, Céline Dard, Philippe Saas, Paolo Cravedi, Zuzana Macek-Jilkova

**Affiliations:** ^1^ Nephrology, Hemodialysis Apheresis and Kidney Transplantation, Department, CHU Grenoble Alpes, Grenoble, France; ^2^ University Grenoble Alpes, CNRS, Inserm, CHU Grenoble Alpes, Institute for Advanced Biosciences, Grenoble, France; ^3^ Department of Medicine, Translational Transplant Research Center, Icahn School of Medicine at Mount Sinai, New York, NY, United States; ^4^ Virology Department, University Hospital Grenoble, Grenoble, France; ^5^ EFS, Recherche et Développement, Grenoble, France; ^6^ Hepato-Gastroenterology and Digestive Oncology Department, CHU Grenoble Alpes, Grenoble, France

**Keywords:** donor-specific antibody, desensitization, kidney transplantation, metabolism, memory B cells, glycolysis

## Abstract

Despite the growing use of desensitization strategies, hyperimmune patients remain at high risk of antibody-mediated rejection suggesting that, even when donor-specific antibodies (DSA) are effectively depleted, anti-donor specific B cells persist. We included 10 highly sensitized recipients that underwent desensitization with plasmapheresis and B cell depletion prior to kidney transplantation. We quantified changes in DSA (luminex), total B-cell subsets (flow cytometry), anti-donor HLA B cells (fluorospot), and single-cell metabolism in serially collected samples before desensitization, at the time of transplant, and at 6 and 12 months thereafter. Desensitization was associated with a decrease in DSA and total memory B cell and naive B cell percentage, while plasma cells and memory anti-donor HLA circulating B cells persisted up to 12 months after transplant. At 12-month post-transplantation, memory B cells increased their glycolytic capacity, while proliferative KI67+ plasma cells modified their metabolism by increasing fatty acid and amino acid oxidation capacity and decreasing their glucose dependence. Despite effective DSA depletion, anti-donor B cells persist in kidney transplant recipients. Due to the reliance of these cells on glycolysis, glycolysis-targeting therapies might represent a valuable treatment strategy.

## Graphical Abstract

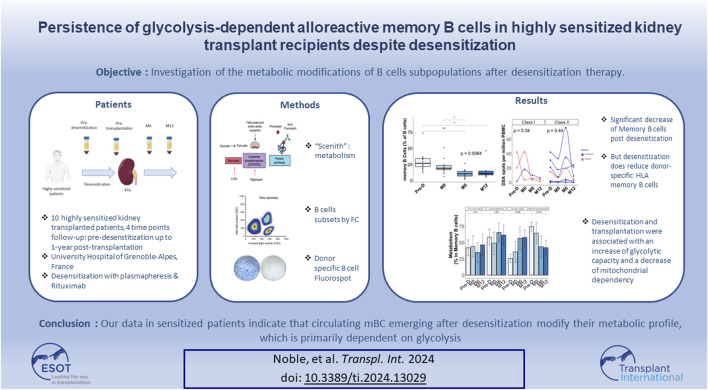

## Introduction

One of the main obstacles to access kidney transplantation (KT) in a context of graft shortage is the presence of anti-human leukocyte antigens (anti-HLA) antibodies. Highly-sensitized patients waiting for a KT represent approximately 10% of the waitlisted individuals and their number is increasing every year [1]. Highly sensitized patient remains on the waiting list longer than non-sensitized patients, despite different national prioritization programs for these patients [2]. This prolonged time on the waiting list and in dialysis is responsible for an increase in patient morbidity and mortality as well as a significant cost to society [3].

To increase the access to transplantation for highly sensitized patients, anti-HLA desensitization may be proposed. Anti-HLA desensitization involves the use of treatments that remove anti-HLA antibodies from plasma and prevent the formation of new anti-HLA antibodies. The most commonly used strategy relies on plasmapheresis and B cell depletion with anti-CD20 monoclonal antibodies (Rituximab) [4–7].

However, despite these techniques that allows KT without hyper-acute rejection, the risk of antibody-mediated rejection (AMR) remains high varying between 15% and 43% depending on the populations, the desensitization techniques, systematic biopsies, and the duration of follow-up [6, 8, 9].

The dynamic changes of B cell compartments, including memory B cells (mBC) after desensitization and post KT are still poorly defined.

Immune cell survival and function are dependent on the adaptability of their metabolism which may be modified by many external factors, including, among many others, inflammation or immunosuppression [10]. Therefore, B cell metabolisms may serve as a surrogate marker for rejection risk. Moreover, metabolic changes of B cells may provide new therapeutic targets to prevent antibody-mediated rejection (AMR) in high-risk patients [11]. This formed the background for the present cohort study aimed at deciphering changes in B cell subsets, including donor-reactive mBCs, and their metabolic profile in highly sensitized kidney transplant recipients undergoing desensitization.

## Materials and Methods

### Patients and Study Design

In this monocentric study, we included highly sensitized (historical PRA ≥85%) adult patients that received a KT at Grenoble University Hospital, Grenoble, France, between January 2015 and November 2022 post-desensitization therapy. Patients had to be on the KT waiting list for at least 3 years before inclusion, to have no history or ongoing severe infectious or neoplastic disease and to have a satisfying cardiac check-up within the previous 3 months. Peripheral Blood Mononuclear Cells (PBMC) were collected at 4 timepoints: before desensitization, the day of transplantation, at 6-month (M6) post-KT and at 12-month (M12) post-KT (Supplementary Figure S1). Donor-specific antibodies (luminex) were quantified at the same timepoints.

The protocol was approved by investigational review board at Grenoble University Hospital (AC-2019-3627) and by French National committee for data protection (CNIL; approval number 1987785v0). All patients signed written informed consent.

### Desensitization Procedure and Post-Transplant Immunosuppression

Desensitization protocol consisted of *i*) two Rituximab i.v., (375 mg/m^2^), 2 weeks apart; *ii*) serial apheresis sessions were performed by immunoadsorption or double-filtration plasmapheresis and a plasma-exchange before the transplantation; *iii*) oral immunosuppression started the first day of first apheresis. The immunosuppression regimen included prednisone (0.5 mg/kg), mycophenolate mofetil (500 mg x2/day), and tacrolimus (0.1 mg/kg/day, then adapted to an 8–10 ng/mL target trough concentration). Post-transplantation, all recipients received anti-thymocyte globulin induction therapy (1 mg/kg/day for 5 days). Tacrolimus and mycophenolate mofetil were continued post-transplantation and steroids were tapered at 3 months post-transplantation (10 mg/day).

### Phenotypic Analysis of B Cell Subpopulations

PBMC were isolated by density gradient centrifugation (Ficoll ^®^) separation and stored at −120°C before use. After thawing, PBMC were incubated at 37°C between 2 and 4 h to allow them recovering from cryopreservation. Dead cells were stained with LIVE/DEAD™- Yellow LDFixable-575 (BD Biosciences) at room temperature during 15 min. Then, PBMC were stained with the mix of fluorochrome-labelled anti-human antibodies during 20 min at 4°C. The panel included following markers: CD20-BV421, CD38-BV510, CD3-BV650, CD14-BV650, CD24-BV711, CD27-BV786, IgM-BB515 and CD19-APC-R718 (BD Biosciences) antibodies.

After washing, cells were fixed and permeabilized using fixation and permeabilization buffer following manufacturer instructions. Intracellular staining was performed by incubating cells during 30 min at 4°C with intracellular antibodies: Puromycin-AF647 (Merck), Ki67-PE and IgD-BB700 (BD Biociences). Stained cells were then directly analysed using the 4-laser BD FACSymphony A3 flow cytometer, data were analysed by BD FACSDiva™ software (BD Biosciences), FCS Express-7 software and Cytobank. A minimum of one million PBMC of cells were used in each experiment. The gating strategy of B cell subpopulations was shown in Supplementary Figure S2.

### Analysis of Cell Metabolism

We used the SCENITH (Single-Cell ENergetIc metabolism by profiling Translation inhibition) technology to assess the metabolic features of B cell subsets at a single cell level [12]. Before antibody staining, PBMC were resuspended in RPMI 10% FCS and were treated during 30 min at 37°C with DMSO (Control), 2DG (100 mM, Sigma), Oligo (1mM, Ozyme), or both (DG + Oligo). Puromycin (Puro, 10 mg/mL, Cayla) was then added during 20 min and cells were washed in cold PBS. The metabolic profiles allowed to distinguish: “glucose dependency” and “mitochondrial dependency” (i.e., the proportion of protein synthesis dependant on glucose and oxidative phosphorylation [OXPHOS], respectively), “glycolytic capacity” showing the maximal capacity of glycolysis to compensate OXPHOS inhibition and “fatty acid and amino acid oxidation (FAO/AAO) capacity” showing FAO/AAO maximal capacity to compensate both glycolysis and OXPHOS inhibition.

### HLA Donor Specific B cells

PBMCs were stimulated at a concentration of 1.5 × 10^6^ PBMC/mL [13, 14]. The non-specific stimulation cocktail consisted in recombinant interleukin 2 (IL-2) at 10 ng/mL and R848 (Resquimod) at 1 μg/mL from Mabtech^®^. After 6 days, cells were added in ELISpot wells at 4.5.10^6^/mL (100 µL) and incubated at 37°C (5% CO_2_) for 24 h in plate coated with anti-human IgG. Anti-HLA specific IgG-antibody secreting cells were detected using fluorescent dye labeled class I and II HLA dextramers and tetramers to cover at least 1 donor specific HLA corresponding to immunodominant DSA in recipients (PE-tagged B51 dextramers, FITC-labelled dextramer A24, PE-labelled dextramer A2) (Immudex^®^, Cy5-labelled DR9 tetramers, Cy3-labelled DQ2 tetramers). FluoroSpot enhancer was added during 15 min before reading. Spot quantification was made on IRIS™ reader (Mabtech^®^). We presented the findings as the proportion of HLA-specific memory B cells (mBC) relative to polyclonal IgG (used as positive controls) for each HLA antigen [15].

### IgG Total Secretion by Antibody Secreting Cells Assessed by B Cell ELISpot With Metabolism Blockade

To assess the effect of glycolysis and OXPHOS blockade on IgG secretion by activated mBC, we performed modified ELISpot protocol. We stimulated PBMC with the same non-specific stimulation cocktail IL-2 at 10 ng/mL and R848 at 1 μg/mL (Mabtech^®^) in addition to 2- DG (100 mM, Sigma), Oligo (1 mM, Ozyme) or both (DG + Oligo). Positive control consisted in Cycloheximide (100 mg/mL, Sigma) and stimulated PBMC cultured without metabolism blockers were used as negative. PBMC were stimulated for 4 days (comparable results *versus* 6 days, data not shown) and added in the anti-IgG coated wells at 4.5.10^4^/mL (100 µL) and incubated at 37°C (5% CO_2_) for 24 h. IgG secretion by polyclonal antibody secreting cells was then revealed colorimetrically after addition of Streptavidin-ALP complex and BCIP/NTB-plus substrate.

### Statistical Analysis

All numerical data were presented as mean ± standard deviation or median [Q1Q3] according to distribution. All categorical variables were presented as number (percentage). Wilcoxon test was used to compare continuous variables and Fisher exact test was used to compare categorical variables. A two-sided *p*-value of <0.05 was considered statistically significant and all *p*-value <0.1 were shown in the figures. To compare the overall differences of subpopulation over time, we first performed an ANOVA. Then we performed two by two analysis between all-time points using *t*-test after Bonferroni correction. Statistical analyses and figures were conducted using R statistical software^®^ 0.98.932 (Boston, MA, USA). Flow cytometry FCS files were analyzed using Cytobank software [16].

## Results

### Patients Baseline Characteristics and Outcomes

We included 10 highly sensitized patients (median PRA: 95% [90.5%–97.0%]) that received a KT post-desensitization at the KT department of Grenoble-Alpes (Grenoble, France). Donor and recipient characteristics are presented in Table 1.

**TABLE 1:** Baseline patients’ characteristics.

	Total n = 10
Donor	
Age (year); median (Q1; Q3)	59.3 ± 11
Gender, male; N (%)	4 (40.0%)
Type, deceased; N (%)	2 (20%)
Recipient	
Age (year); median (Q1; Q3)	48 ± 14
Gender, male; N (%)	4 (40)
Delay between desensitization and transplantation (day); median (IQR)	28.5 (23; 31)
Time on the transplant waiting list (year); median (Q1; Q3)	3.8 (3.0; 5.5)
Initial nephropathy	
- Hypertension	4 (40)
- Diabetes	2 (20)
- ADPKD	1 (10)
- Membranous nephropathy	1 (10)
Unknown	2 (20)
Graft number, first; N (%)	6 (60)
Sensitizing events; N (%)	
- Pregnancy	5 (50)
- Blood transfusion	3 (30)
- Transplantation	4 (40)
Panel Reactive Antigen; median (Q1; Q3)	95 (90.5; 97.0)
Mismatch AB; median (Q1; Q3)	3.0 (2.0; 3.0)
- 1; N (%)	2 (20)
- 2; N (%)	2 (20)
- 3; N (%)	5 (50)
- 4; N (%)	1 (10)
Mismatch DQ; median (Q1; Q3)	1.5 (0.0; 2.0)
- 0; N (%)	4 (40)
- 1; N (%)	1 (10)
- 2; N (%)	5 (50)
Mismatch DR; median (Q1; Q3)	0.5 (0.0; 1.5)
- 0; N (%)	5 (50)
- 1; N (%)	3 (30)
- 2; N (%)	2 (20)
Crossmatch positivity on historical sera; N (%)	2 (20)
- LCT	3 (30)
- FACS	5 (50)
Desensitization protocol; N (%)	
- RTX + maintenance therapy + plasmapheresis	9 (90)
- RTX + maintenance therapy	1 (10)
Plasmapheresis type; N (%)	
- PE	3 (30)
- DFPP	6 (60)
- IA	4 (40)
Number of plasmapheresis session; median (Q1; Q3)	8 (7; 12)
Induction therapy, ATG; N (%)	10 (100)
Maintenance therapy, Tac MMF Cs; N (%)	10 (100)

ADPKD, Autosomal dominant polycystic kidney disease LCT, lymphocytotoxicity cross-math; FACS, Flow-cytometry cross-match; RTX, rituximab; PE, plasma exchange; DFPP, double-filtration plasmapheresis; IA, immunoadsorption; ATG, anti-thymoglobulin; Tac, Tacrolimus; MMF, mycophenolate mofetil; CS, corticosteroids.

**FIGURE 1:**
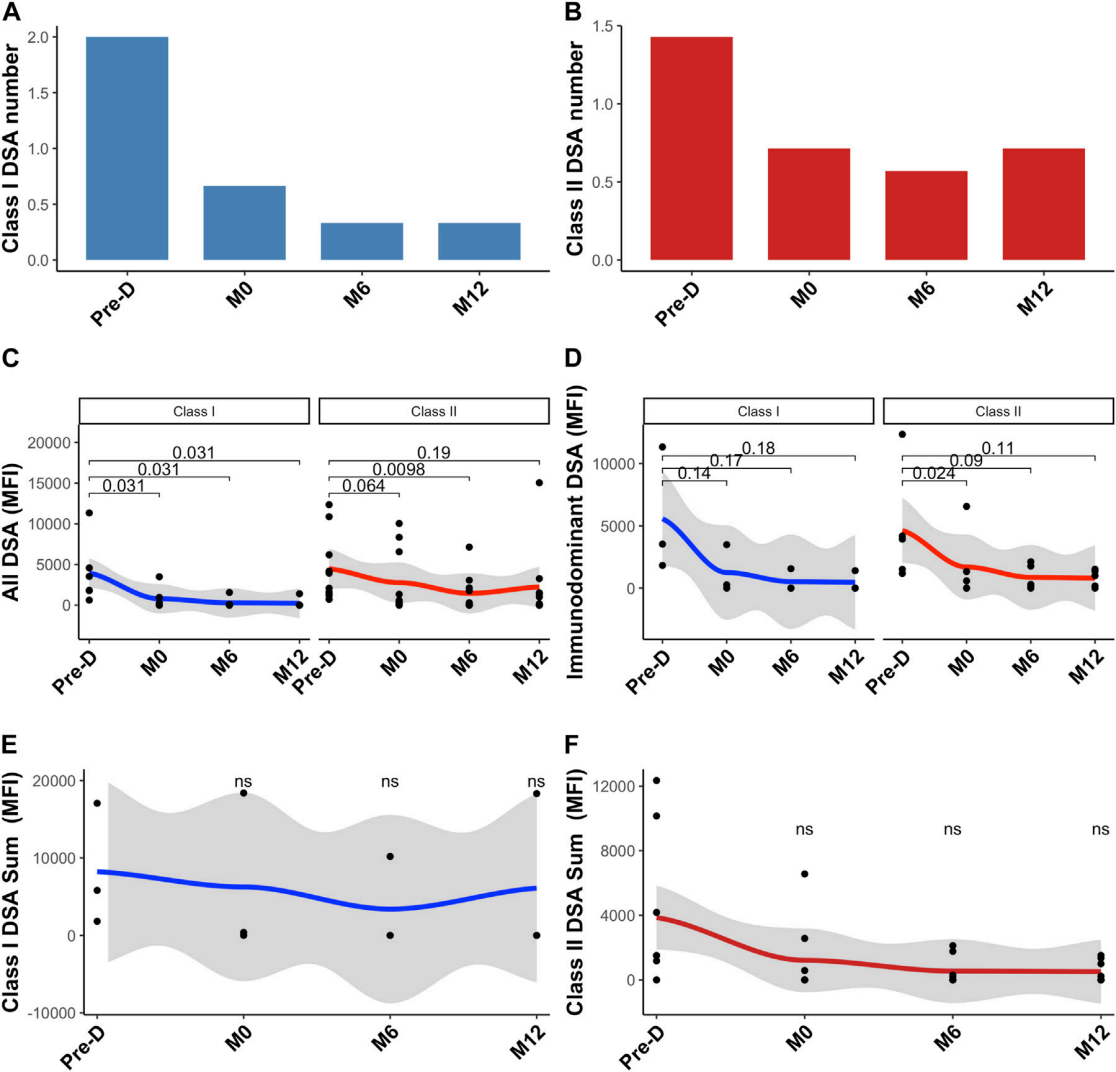
Donor-specific antibodies change post desensitization and transplantation. Trends in the number of class I DSA **(A)**, class II DSA **(B)**, in all class I and II DSA MFI **(C)**, in immunodominant class I and class II DSA **(D)**, in class I sum of MFI **(E)** and in class II sum of MFI **(F)**. Blue is for class I DSAs and Red is for class II DSAs. Pre-D: pre-desensitization.

Before desensitization, mean number of class I DSA was 2.0 ± 1.0 and mean number of class II DSA was 1.4 ± 0.8. The day of KT, mean number of class I DSA was 0.7 ± 1.2 and mean number of class II DSA was 0.6 ± 0.5 (Figures 1A, B). Desensitization therapy was associated with a significant decrease in all DSA MFI and allowed KT with negative crossmatch (Figure 1C). Mean immunodominant MFI (Mean Fluorescence Intensity) for class I DSAs before desensitization was 5,564 ± 5,072 and decreased to 1252 ± 1943 the day of transplantation, *p* = 0.14 (Figure 1D). Mean immunodominant class II DSA MFI before desensitization was 4629 ± 526 and decreased to 1694 ± 2774 the day of transplantation, *p* = 0.024 (Figure 1D). The sum of all class I MFI was 8,232 ± 7,903 before desensitization and 6,102 ± 10,570 at 12-month, *p* = 0.507 (Figure 1E) and all class II MFI was 4195 ± 5058 before desensitization and was 586 ± 681 at 12-month, *p* = 0.170 (Figure 1F).

At the end of the follow-up period (61.6 months [49–68]), all patients were alive and none of them had lost their graft. Three patients developed acute rejection: two patients developed AMR and one patient had a cellular borderline rejection. All patients were successfully treated with steroids pulse and plasmapheresis. Although non statistically significant, immunodominant DSA mean MFI was higher in patients with rejection as compared with those without rejection (8,503 ± 5,808 versus 2,866 ± 1,413.8, *p* = 0.180). Similarly, sum of all DSA MFI was higher in patients with rejection: 12,776 ± 11,173 as compared to those without rejection: 3,619 ± 4,116 (*p* = 0.086).

### Changes in B Cells Subsets

**FIGURE 2:**
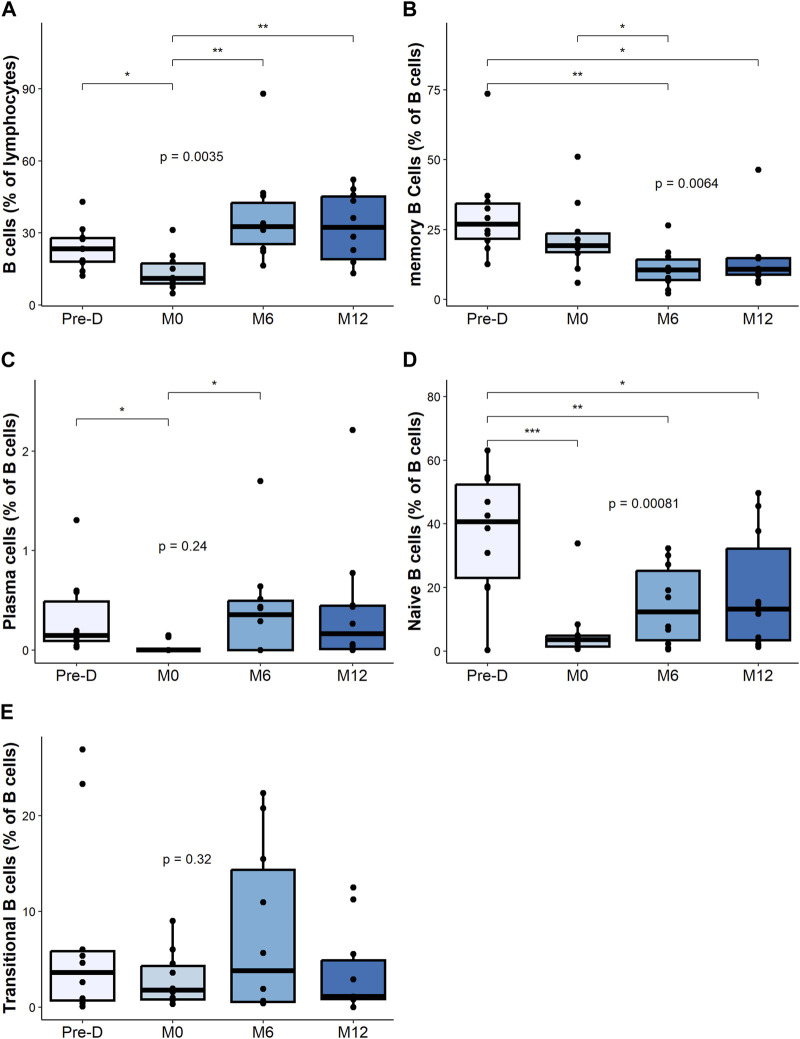
B cell composition and change post desensitization and transplantation. Trends in B cells and plasma cells before and after desensitization and transplantation: total B cells (CD19^+^CD3^−^CD14^−^) **(A)**, memory B cells (CD19^+^CD38^−^ CD24^+^ CD27^+^) **(B)**, plasma cells (CD19^+^CD27^hi^ CD38^hi^) **(C)**, naïve B cells (CD19^+^CD27^–^ IgD^+^) **(D)**. Transitional B cells (CD19^+^ CD27^–^ CD24^hi^) **(E)**. *p < .05 by Anova test. PBMC were also available for all 10 patients at the 4 timepoints.

Upon rituximab therapy, CD19^+^ B cells were effectively depleted and fully recovered at 6 months post-transplant (Figure 2A). The percentage of memory B cells (mBC) significantly declined after desensitization and did not fully recover post-transplantation (Figure 2B).

The frequency of plasma cells decreased, but not significantly with desensitization therapy. We observed a return to baseline level at 6 months and at 12 months after transplantation (Figure 2C).

We observed a marked decrease of the naive B cell (CD27^−^ IgD^+^) after desensitization that did not fully recover post-transplantation (Figure 2D). The frequency of transitional B cells did not significantly change after desensitization (Figure 2E).

We found no correlation between DSA and anti-HLA specific B cells.

### Changes in Proliferative B Cell Subsets

**FIGURE 3:**
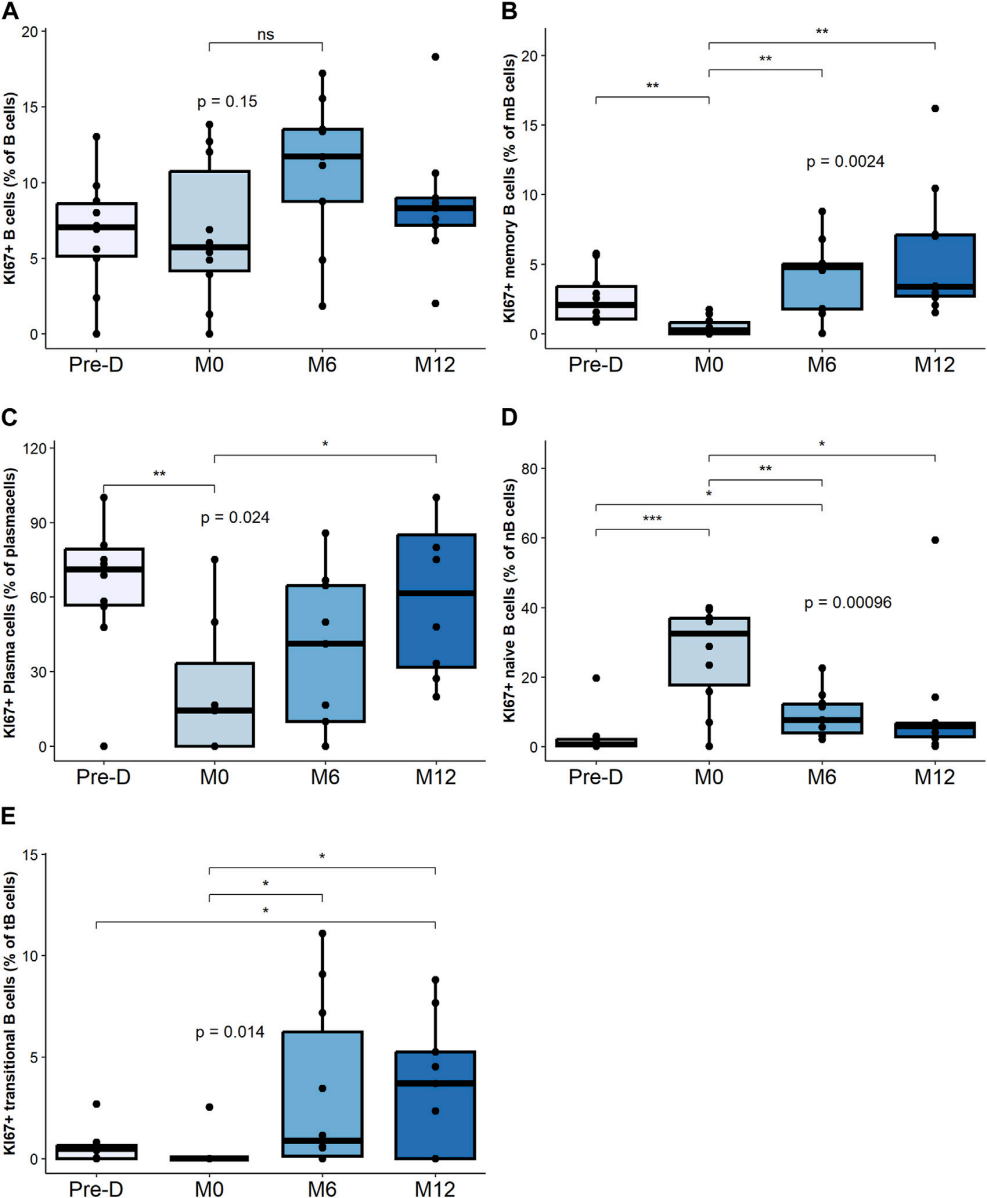
Proliferative B cell composition and change post desensitization and transplantation. Trends in KI67 + B cells and plasma cells before and after desensitization and transplantation: total B cells (CD19^+^CD3^−^CD14^−^) **(A)**, memory B cells (CD19^+^CD38^−^ CD24^+^ CD27^+^) **(B)**, plasma cells (CD19^+^CD27^hi^ CD38^hi^) **(C)**, naïve B cells (CD19^+^CD27^–^ IgD^+^) **(D)**. Transitional B cells (CD19^+^ CD27^–^ CD24^hi^) **(E)**. *p < .05 by Anova test. PBMC were also available for all 10 patients at the 4 timepoints.

While the relative percentage of total Ki67^+^ B cells was not impacted by desensitization and transplantation (Figure 3A), desensitization was associated with a decrease of Ki67^+^ mBC percentage, followed by an increase at M6 and M12 (Figure 3B).

The percentage of Ki67^+^ plasma cells significantly decreased post-desensitization and recovered to pre-desensitization levels at M12 post-transplant (Figure 3C).

The percentage of Ki67^+^ naïve B cells, significantly increased post-desensitization and decreased post-transplantation (Figure 3D). The percentage of Ki67^+^ transitional B cells progressively increase after transplant increased up to M12 (Figure 3E).

Proliferative mBC and transitional B cells are the subsets that increased the most after transplant.

### Desensitization Does Not Reduce Donor-Specific Anti-HLA B Cells

**FIGURE 4:**
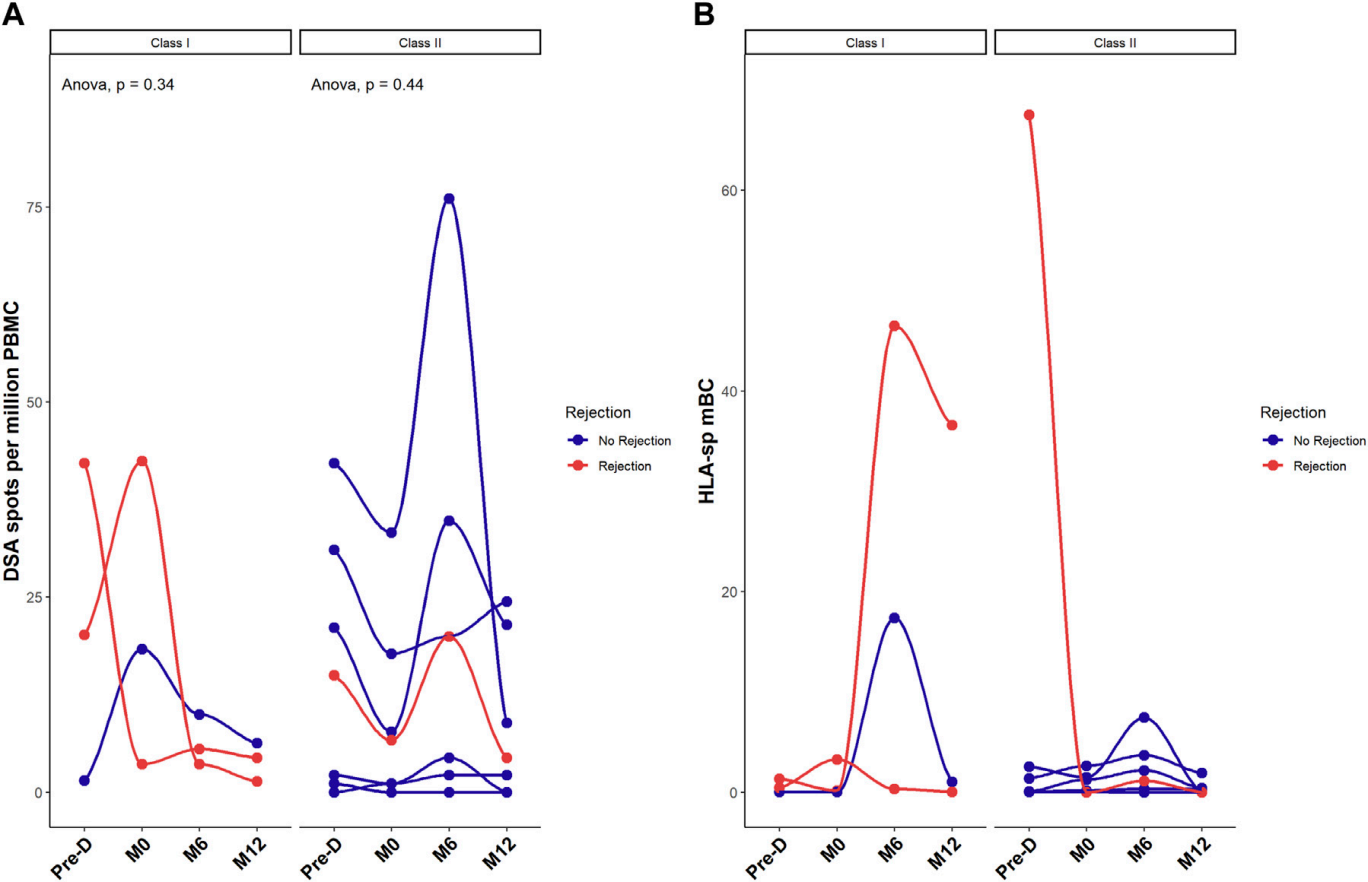
Desensitization does reduce donor-specific HLA memory B cells. Donor-specific memory B cell differentiation into plasma cells assessed by FluoroSpot measuring donor-specific antibodies (spots) and expressed as mean number of spots per million of PBMC **(A)** and ratio between HLA-specific mBCs over the total polyclonal IgG mBCs in each patient **(B)**. Red dots correspond to patients with rejection. *p < .05 by Anova test. PBMC were also available for all 10 patients at the 4 timepoints.

Next, we focused on the donor-specific anti-HLA B cells by FluoroSpot. The number of total IgG spots secreted by polyclonal antibody secreting cells was positively correlated (*p* = 0.012) with the percentage of mBC assessed by Flow cytometry (Supplementary Figure S3) but not with the other B cells subtypes (data not shown). Median number of donor specific anti-HLA B cells was 17.6 spots [1.7–28.6] before desensitization and 7.2 spots [1.7–18.2] at pre-transplantation, *p* = 0.41. This secretion remained stable at 6 months: 7.8 spots [3.8–20] and at 12 months: 4.4 spots [1.6–8.2] post-transplantation. Figure 4 shows the evolution of class I and class II donor-specific anti-HLA B cells. There was no statistical difference between patients with rejection and those without rejection regarding the evolution of donor-specific anti-HLA B cells.

Three patients developed rejection post transplantation. The number of donor-specific anti-HLA IgG was higher in those three patients at pre-desensitization time as compared to the recipients without humoral rejection although this difference did not reach significance: 26 ± 14 spots in patients with rejection, versus 14 ± 17 spots in recipients without rejection, *p* = 0.37 (despite a significantly higher total IgG secretion in patients without rejection: 4950 spots per million of PBMC [999–17,238] *versus* 2475 [876–25,825] in patients with AMR, *p* < 0.001). Supplementary Figure S4 illustrates the stereotypical evolution profile of DSA in relation to similar HLA-specific mBC as measured by FluoroSpot in 4 patients. This data shows that there is no clear correlation between the levels of DSA and the frequency of donor-specific mBC.

### Changes in B Cell Metabolic Profile

Before desensitization, the metabolic profile was similar across all B subpopulations (Supplementary Figure S5A), were also not statistically different at 12 months post-post-transplantation (M12) (Supplementary Figure S5B).

**FIGURE 5:**
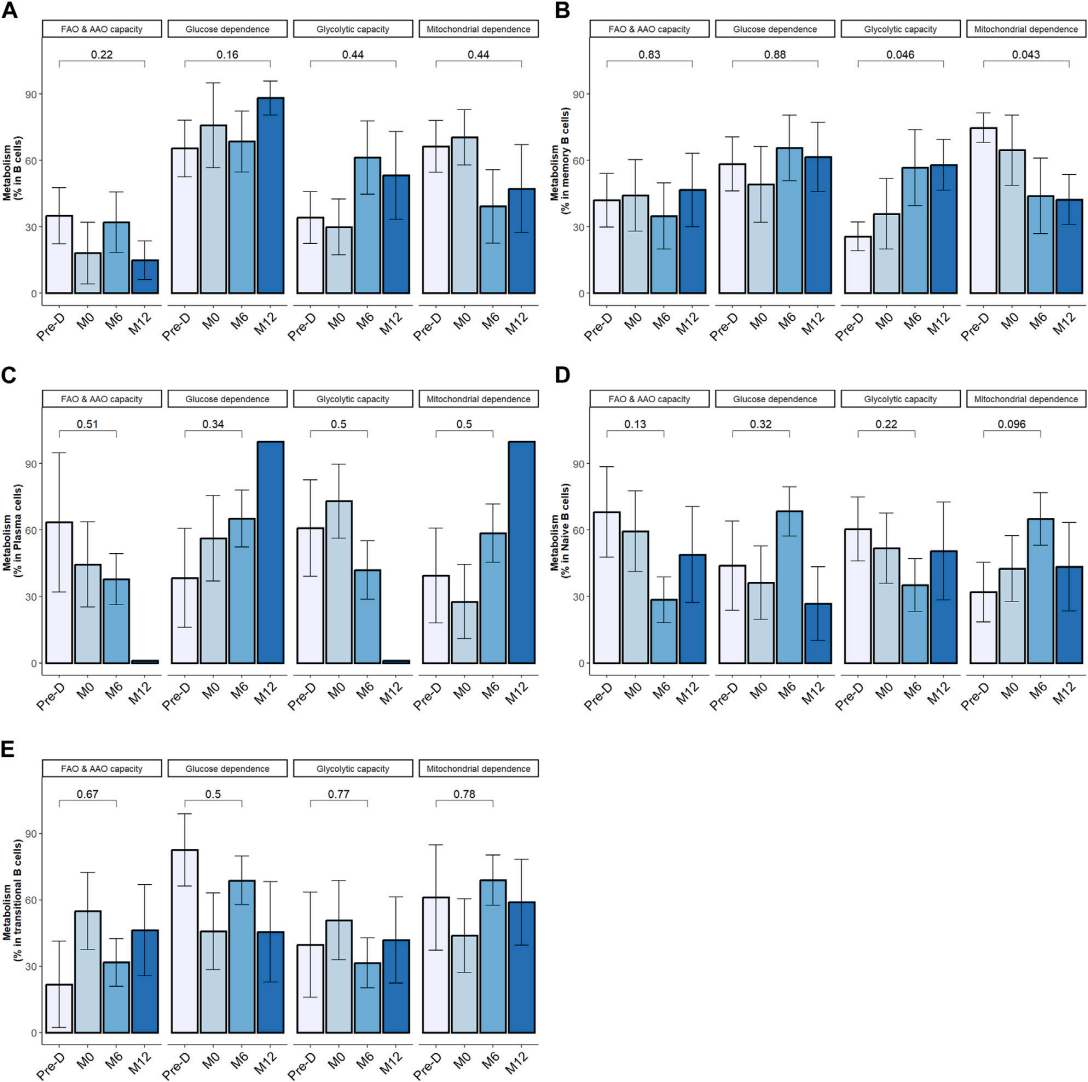
Desensitization and transplantation impact on B cell metabolism. Trends in B cell subsets metabolism profile evolution before desensitization (Pre-D), pre-transplantation (M0), at month-6 (M6) and at month-12 (M12) post-transplantation; total B cells (CD19^+^CD3^−^CD14^−^) **(A)**, memory B cells (CD19^+^CD38^−^ CD24^+^ CD27^+^) **(B)**, plasma cells (CD19^+^CD27hi CD38hi) **(C)**, naïve B cells (CD19^+^CD27^−^ IgD^+^) **(D)**. Transitional B cells (CD19^+^ CD27^−^ CD24hi) **(E)**. The p-value is indicated the evolution of metabolism percentage between Pre-D and M12. *p < .05 by Wilcoxon test. FAO: fatty acids oxidation; AAO: amino acids oxidation. 4 patients had 1 timepoint missing.

Within the overall B cell population, desensitization and transplantation had no significant impact on cell metabolism (Figure 5A). We next zoomed in on B cell subpopulations metabolism.

**FIGURE 6:**
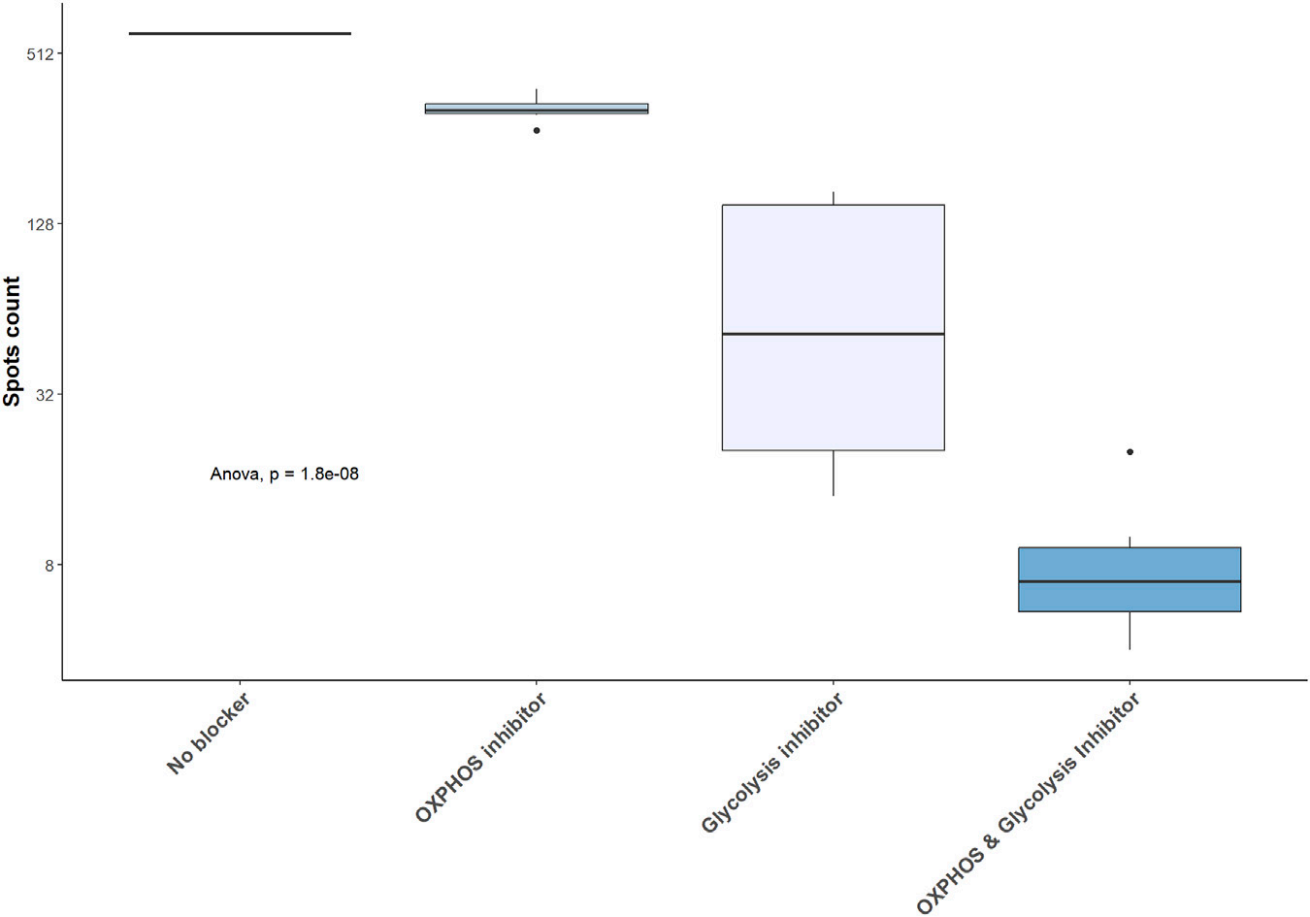
Glycolysis inhibition results in a significant decrease in IgG secretion by activated memory B cell. IgG secretion assessed by ELISpot in 5 kidney transplant recipients and expressed as raw number of spots. OXPHOS: oxidative phosphorylation.

In mBC, desensitization and transplantation were associated with an increase of glycolytic capacity between pre-desensitization and M12 and a decrease of mitochondrial dependency (Figure 5B). FAO/AAO capacity and glucose dependence were not impacted. To further define the dependency of mBC in kidney transplant recipients on glycolysis, we performed ELISpot with different metabolism inhibitors. Selective inhibition of glycolysis resulted in a significant decrease of IgG secretion (spots) by activated memory B cells, while OXPHOS inhibition did not affect it (Figure 6).

Metabolic requirements did not significantly change over follow-up period in naïve B cells, transitional B cells and plasma cells (Figures 5C–E). In KI67+ plasma cells, percentage of FAO/AAO capacity significantly increased post desensitization and transplantation while glucose dependence percentage significantly decreased (Supplementary Figure S6). The metabolic modifications were not statistically significant in KI67+ mBC. The tendency was similar with a median increase of glycolytic capacity from 26% at baseline to 60% at M12, *p* = 0.44 and a median decrease of mitochondrial dependence from 73% at baseline to 31% at M12.

## Discussion

Previous studies have shown a clear correlation between circulating HLA-specific mBCs and high risk of antibody-mediated acute and chronic rejection in kidney transplantation [14, 15]. However, no study has formally investigated the changes in circulating HLA-specific mBCs after desensitization. Our data indicate that apheresis and B cell depletion, together with chronic immunosuppression are effective in removing DSA allowing for transplantation procedure, but do not clear HLA-specific mBCs. These cells are mainly located in peripheral lymphoid organs and, upon re-encounter with target antigens, can differentiate into antibody-secreting cells [17]. Therefore, they may account for the high risk of ABMR despite effective DSA removal after desensitization [18].

Long-lived plasma cells are also a major source of alloantibodies. These cells reside primarily in the bone marrow where they continuously secrete antibodies [19]. Our data indicate that desensitization and immunosuppression are not able to reduce circulating plasma cells but reduced the proliferative ratio of plasma cells at the initial phase. A limitation of our study lies in the fact that we only analyzed circulating plasma cells and long-lived plasma cells reside in the bone marrow. Some data on long-lived plasma cells suggest that their metabolism requires more glucose and amino acids than short-lived plasma cells (ref lam cell rep). No study has reported on the metabolic changes induced by allostimulation of long-lived plasma cells.

Metabolism has been shown to shape the survival and functionality of innate and adaptive immune cells [20, 21]. However, metabolic profile of B cells has been poorly characterized, especially in the field of solid-organ transplantation. We observed that, in sensitized patients, B cell subsets have a similar baseline metabolism profile characterized by a high glucose and mitochondrial dependency associated with a lower level of FAO, AAO and glycolytic capacities. Interestingly, after desensitization and after transplantation, we observed different metabolic modifications within the B cell subpopulations. After desensitization, total B cells re-emerged to baseline level but with more heterogenicity in their metabolism capacities. mBC percentages did not fully recover after transplant. Of note, those that formed after desensitization had a high glucose dependency, higher glycolytic capacity, and lower OXPHOS metabolism than at baseline. Interestingly, Torigeo *et al.* showed that glucose uptake and glycolysis are important for mBC differentiation into plasma cells [22]. This may at least in part explain the glycolytic capacity increase in mBC post-KT of sensitized kidney recipients and the major impact of antibody secretion by glycolysis inhibitor in the ELISpot results. After desensitization and transplantation, we also observed a re-emergence of proliferative plasma cells with a different metabolic profile, i.e., with higher FAO/AAO capacity and less glucose dependence. This is consistent with Lam *et al.* showing an elevated expression of an amino-acid transported in long-lived plasma cells [23].

Improvement in immunosuppressive strategies have contributed to improve long-term patient and graft survival. Yet, long-term immunosuppression is burdened by increased risk of infections, cancer, and metabolic complications [24–26]. Targeting metabolism, especially by blocking specific pathways may effectively control alloimmune response.

We tried to directly modify the metabolism of antibody-secreting cells using glycolysis and OXPHOS inhibitors. To date, direct modulation of immune cell metabolism has only been assessed in innate immune cells and T lymphocytes [27, 28]. The authors showed that, the adjunction of metabolism inhibitors (glycolysis inhibitor and glutamine inhibitor) on top of immunosuppression, increases skin and heart allograft survival in a mice model [29, 30].

Several limitations exist in our study. Firstly, the sample size of included patients is relatively small. This limitation stems from the infrequent occurrence of desensitization procedures within our patient population. From a technical point of view, cryopreservation may impact the results of metabolism assessment. To minimize this potential confounder, we allowed cells to recover in the incubator for a short period before doing the analyses [31, 32]. However, despite this constraint, our cohort exhibits comprehensive phenotypic characterization. Additionally, we were able to analyze serial samples from all participants, allowing for thorough investigation. Our investigation focused on analyzing B cell metabolism, yet numerous other cell subtypes play crucial roles in the cascade of allograft rejection and merit deeper examination. Recent literature highlights the significance of glycolysis in macrophages that infiltrate the graft (REF transplant). Additionally, metabolic pathways like the polyamine pathway have been implicated in modulating Th17 pathogenicity (ref COMP). There remains ample scope for elucidating the immune-metabolism nuances specific to each cell subtype within the realm of solid-organ transplantation and rejection.

Our data in sensitized patients indicate that circulating mBC emerging after desensitization modify their metabolic profile, which is primarily dependent on glycolysis. Therefore, targeting this pathway specifically in mBC may represent a valuable therapeutic option to deplete mBCs, avoid the antibody rebound and maybe reduce the risk of AMBR.

## Data Availability

The raw data supporting the conclusions of this article will be made available by the authors, without undue reservation.

## Abbreviations

FAO/AAO, fatty acid and amino acid oxidation; HLA, Human Leukocyte Antigen; IL-2, interleukin 2; mBC, memory B cells; Oligo, Oligomycin; OXPHOS, Oxidative phosphorylation; PBMC, Peripheral Blood Mononuclear Cells; SCENITH, Single-Cell ENergetIc metabolism by profiling Translation inhibition; 2-DG, 2-deoxyglucose.
