# Corrigendum: Glycolysis Changes in Alloreactive Memory B Cells in Highly Sensitized Kidney Transplant Recipients Undergoing Desensitization Therapy

**DOI:** 10.3389/ti.2024.13876

**Published:** 2024-11-04

**Authors:** Johan Noble, Lara Cabezas, Aurelie Truffot, Lucile Dumolard, Thomas Jouve, Paolo Malvezzi, Lionel Rostaing, Céline Dard, Philippe Saas, Paolo Cravedi, Zuzana Macek-Jilkova

**Affiliations:** ^1^ Nephrology, Hemodialysis Apheresis and Kidney Transplantation, Department, CHU Grenoble Alpes, Grenoble, France; ^2^ University Grenoble Alpes, CNRS, Inserm, CHU Grenoble Alpes, Institute for Advanced Biosciences, Grenoble, France; ^3^ Department of Medicine, Translational Transplant Research Center, Icahn School of Medicine at Mount Sinai, New York, NY, United States; ^4^ Virology Department, University Hospital Grenoble, Grenoble, France; ^5^ EFS, Recherche et Développement, Grenoble, France; ^6^ Hepato-Gastroenterology and Digestive Oncology Department, CHU Grenoble Alpes, Grenoble, France

**Keywords:** donor-specific antibody, desensitization, kidney transplantation, metabolism, memory B cells, glycolysis

In the published article, there was an error in the **Article title**. Instead of “Glycolysis Changes in Alloreactive Memory B Cells in Highly Sensitized Kidney Transplant Recipients Undergonig Desensitization Therapy,” it should be “Glycolysis Changes in Alloreactive Memory B Cells in Highly Sensitized Kidney Transplant Recipients Undergoing Desensitization Therapy.”

The authors apologize for this error and state that this does not change the scientific conclusions of the article in any way. The original article has been updated.

