# Supplementary material for: Glycolysis Changes in Alloreactive Memory B Cells in Highly Sensitized Kidney Transplant Recipients Undergoing Desensitization Therapy
Source: Transpl Int. 2024 Jul 8;37:13029. doi: 10.3389/ti.2024.13029 (PMC11287219; doi:10.3389/ti.2024.13029)
Supplement: Supplementary file 1 [file DataSheet1.PDF]

Capsule Sentence Summary: Our study identifies metabolic shifts in B cells post-transplantation, highlighting glycolysis as a potential target for preventing antibody-mediated rejection in kidney transplant recipients.

Figure S1: Design of the study.

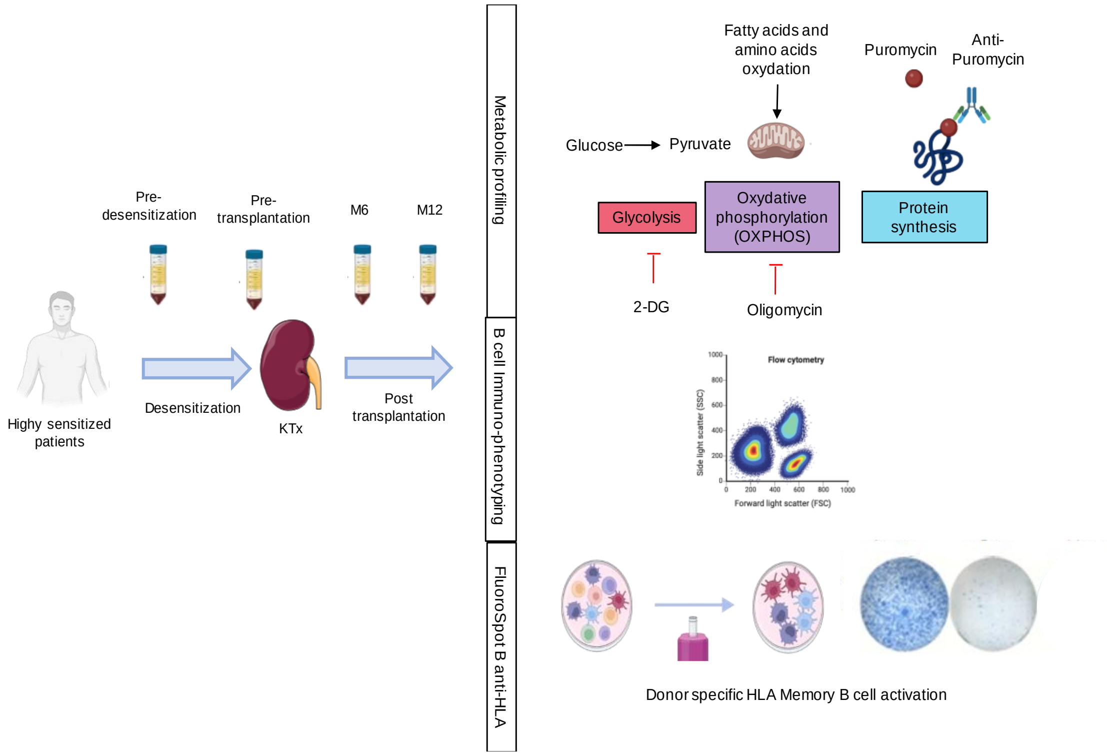

Peripheral Mononuclear cells (PBMC) were retrieved in highly sensitized patients undergoing desensitization to access kidney transplantation. PBMCs were collected before desensitization (Pre-D), at pre-transplantation (M0), at month 6 (M6) post-transplantation and at month 12 (M12) post-transplantation. Simultaneously, PBMCs were used to analyze B cell subpopulations by immuno-phenotyping, metabolic profiling by SCENITH method and HLA B cell FluoroSpot.

Figure S2: Gating strategy of B cells.

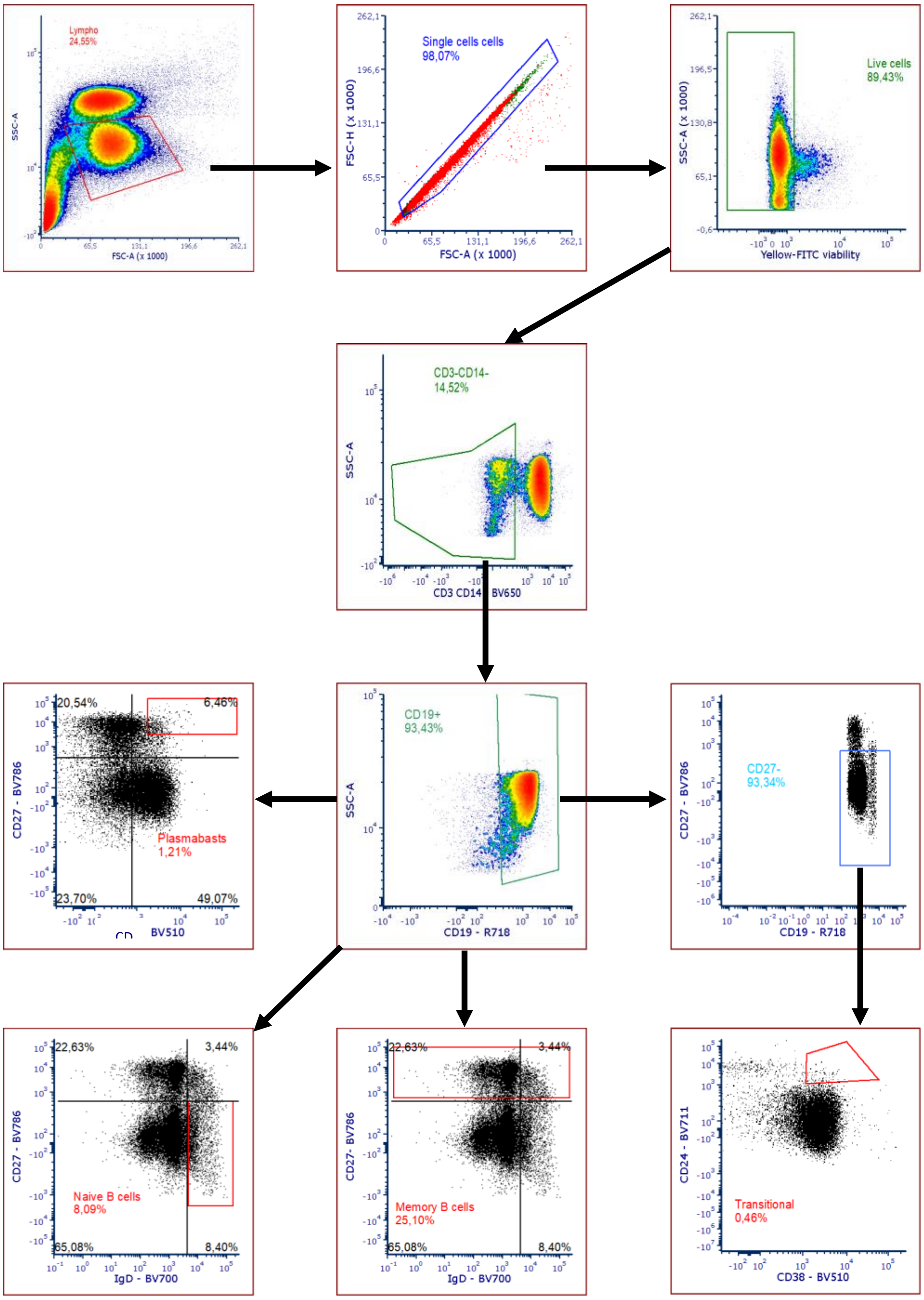

Mononuclear cells were gated out of all events followed by single cell gating and live cells. We then used a dumped channel to exclude T lymphocytes ($CD3^+$) and most of monocytes ($CD14^+$) to improve our sensitivity. B cells were then gated as $CD19^+$ cells. Finally, B cells were subdivided into plasma cells ($CD38^{hi}CD27^{hi}$), naive B cells (IgD^+CD27^-), memory B cells ($CD27^+$) and transitional B cells ($CD27^-CD24^{hi}CD38^{hi}$).

Figure S3: Correlation between total IgG spots secreted by polyclonal antibody secreting cells by FluoroSpot and percentage of memory B cells

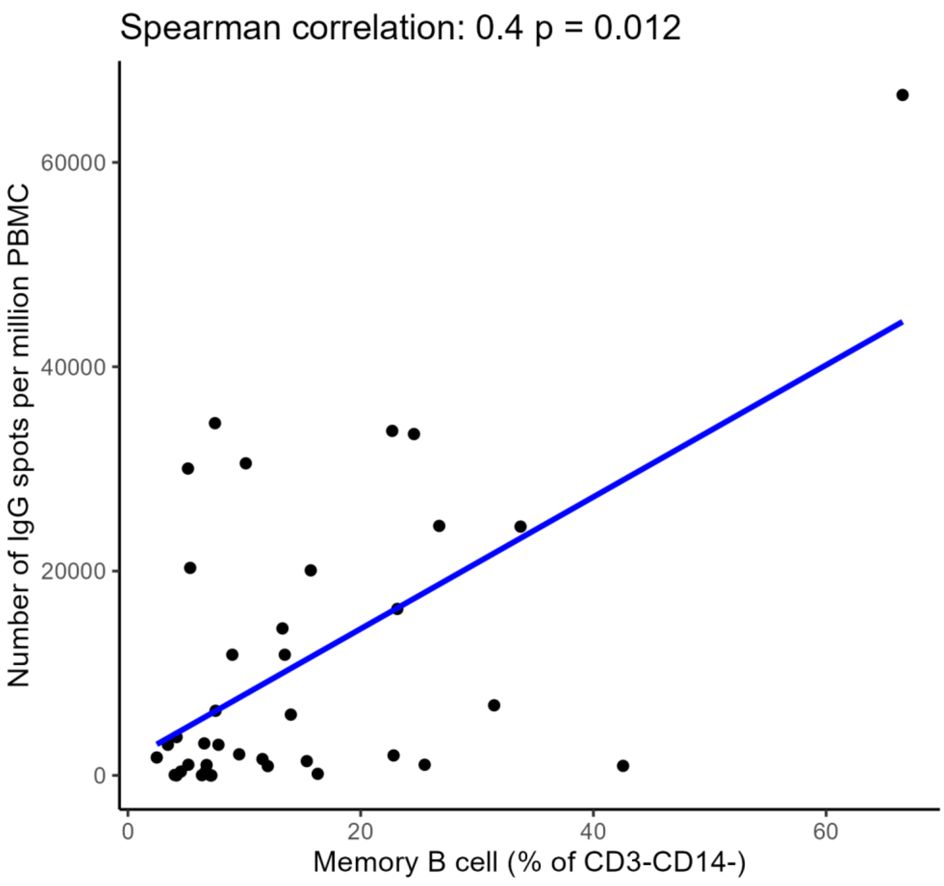

Memory B cells are gated CD27^{+} within CD3- and CD14- lymphocytes. Memory B cell differentiation into plasma cells assessed by FluoroSpot measuring total IgG spots secreted by polyclonal antibody secreting cells and expressed as mean number of spots per million of PBMC. All analysis times were included, i.e. pre-desensitization, pre-transplantation, M6 and M12 post-transplantation.

Figure S4: Correlation between antigen specific DSA and memory B cell by FluoroSpot

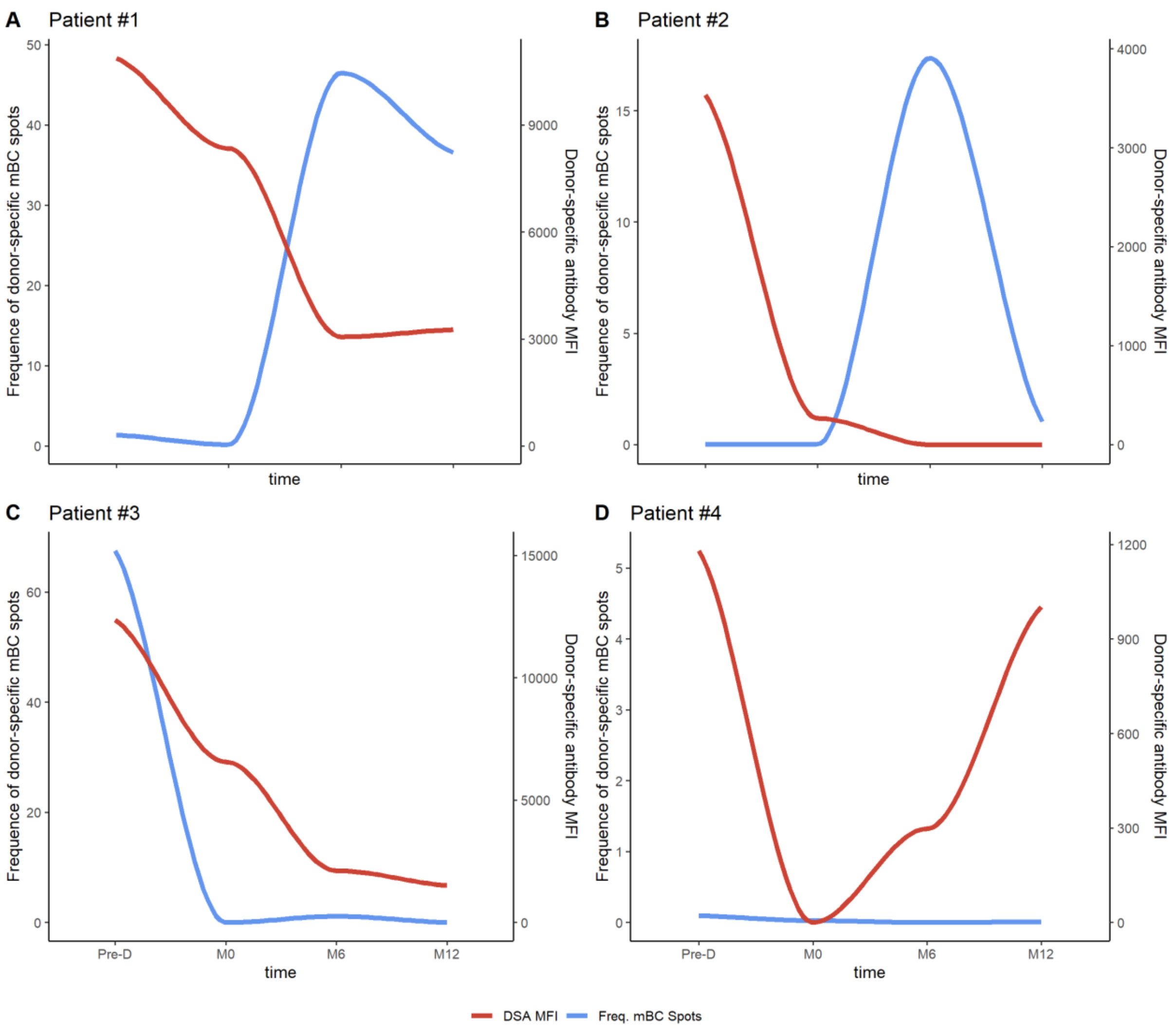

Donor-specific Memory B cell (mBC) differentiation into plasma cells are assessed by Fluorospot (blue line). Donor-specific Antibody (DSA) of the same HLA antigen are quantified using Luminex assay (red line). Patient #1 and Patient #3 experienced acute antibody-mediated rejection in the follow-up of transplantation. Three patients has a similar pattern as patient #3 (i.e. decreasing of DSA and specific mBC without any rebound) and 3 similar du patient #2 (i.e. rebound of specific mBC without the rebound of DSA). Patient #1 showed an important increase of specific mBC associated with a less efficient decrease of DSA post transplantation, MFI > 3000).

Figure S5: Metabolic profile of B cells at baseline and at 12-months.

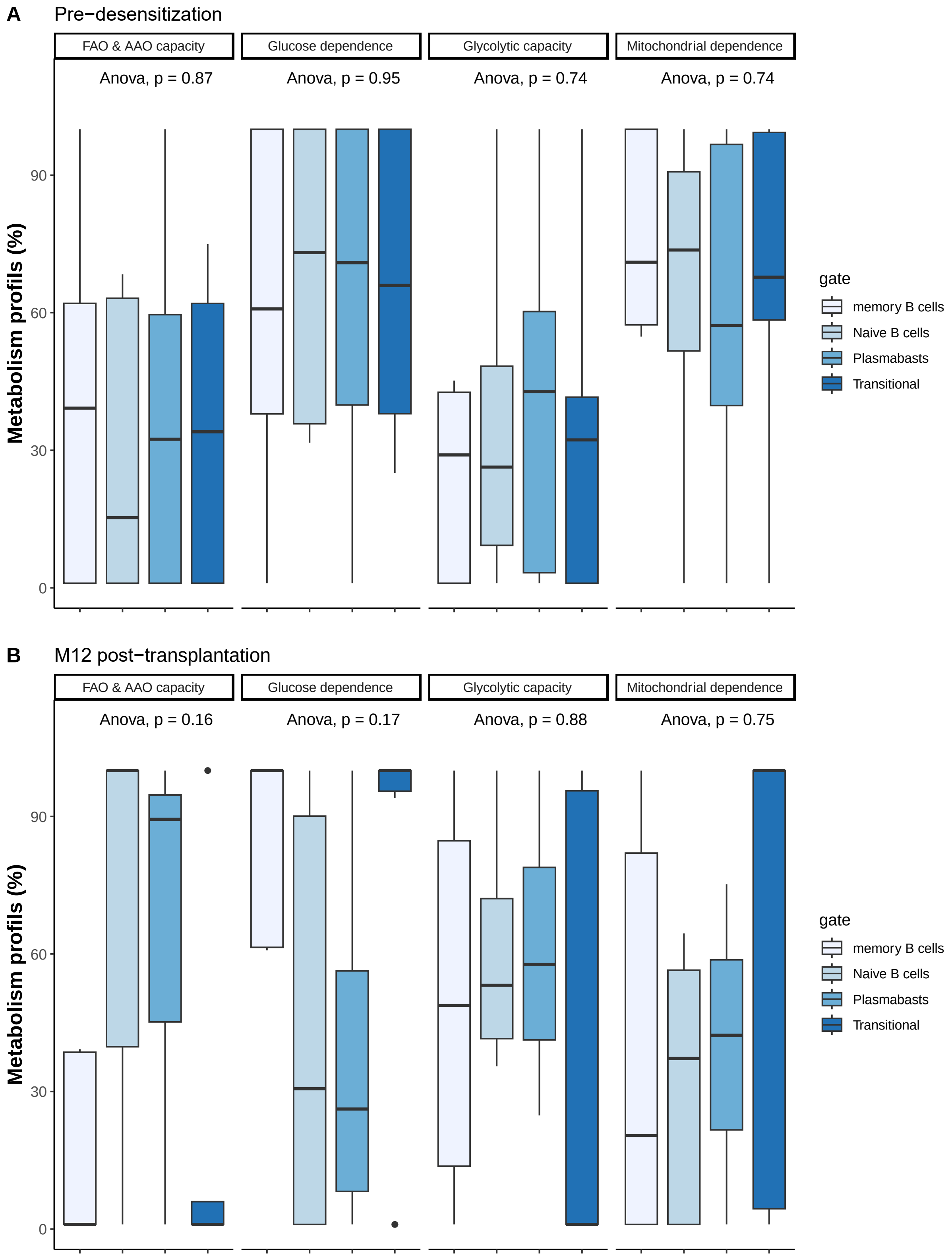

Boxplots of metabolism profile (%) in each B cells subtypes before desensitization (Pre-D), and at month-12 (M12) post-transplantation. *P < .05 by Anova test. FAO: fatty acids oxidation; AAO: amino acids oxidation.

Figure S6: Desensitization and transplantation impact on KI67+ plasma cells.

Figure S5

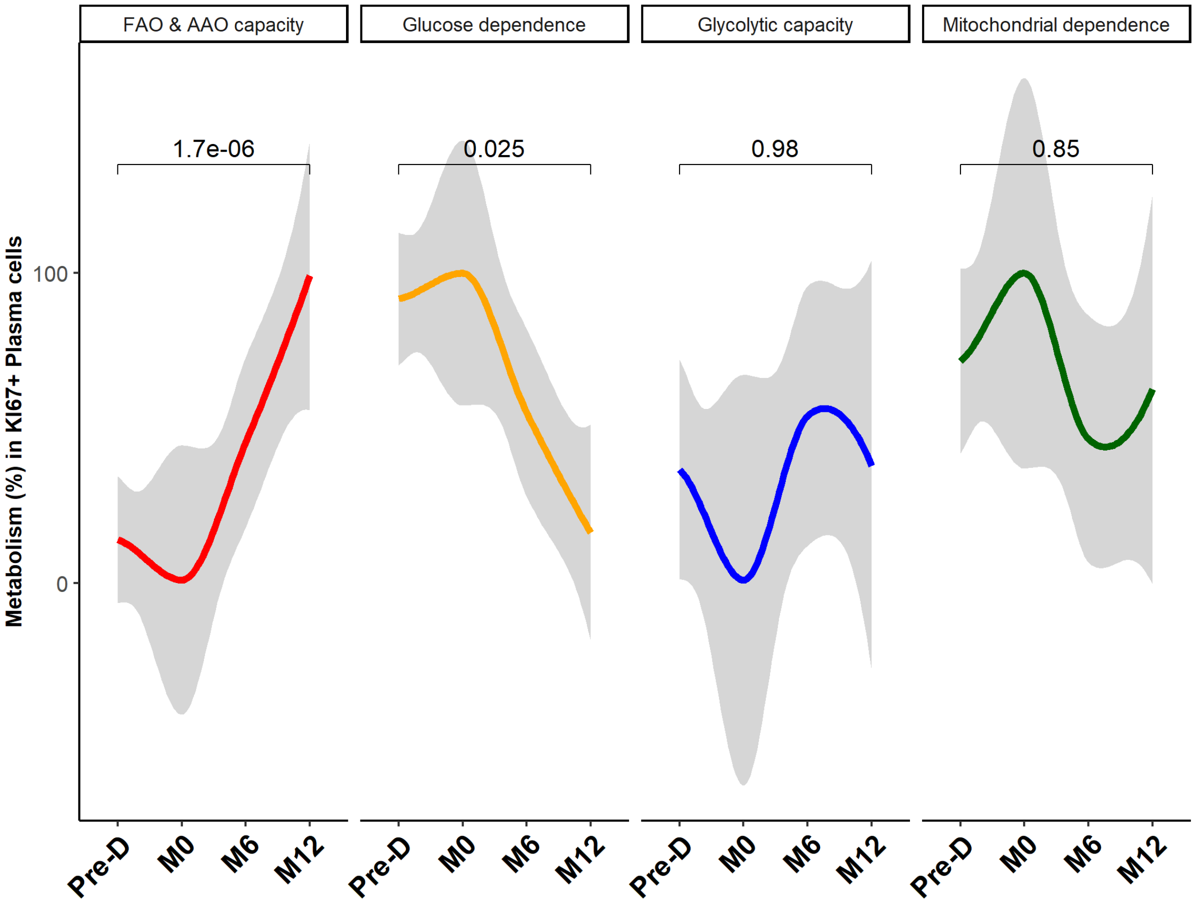

Trends in KI67+ plasma cells metabolism profile evolution before desensitization (Pre-D), pre-transplantation (M0), at month-6 (M6) and at month-12 (M12) post-transplantation. The P value is indicated the evolution of metabolism percentage between Pre-D and M12. *P < .05 by Wilcoxon test. FAO: fatty acids oxidation; AAO: amino acids oxidation.
